# Peritoneal metastases from rare ovarian cancer treated with cytoreductive surgery and hyperthermic intraperitoneal chemotherapy (CRS/HIPEC)

**DOI:** 10.1515/pp-2023-0019

**Published:** 2023-12-27

**Authors:** Luis Felipe Falla-Zuniga, Armando Sardi, Mary Caitlin King, Felipe Lopez-Ramirez, Philipp Barakat, Carol Nieroda, Teresa Diaz-Montes, Vadim Gushchin

**Affiliations:** Surgical Oncology, Institute for Cancer Care at Mercy, Mercy Medical Center, Baltimore, MD, USA; Gynecological Oncology, The Lya Segall Ovarian Cancer Institute, Mercy Medical Center, Baltimore, MD, USA

**Keywords:** peritoneal neoplasms, cytoreduction surgical procedures, hyperthermic intraperitoneal chemotherapy, ovarian neoplasms

## Abstract

**Objectives:**

There are limited treatment options and no consensus on the management of advanced rare ovarian malignancies. Rare ovarian malignancies can present with peritoneal metastases (PM), featuring a similar presentation to more common ovarian subtypes. Cytoreductive surgery and hyperthermic intraperitoneal chemotherapy (CRS/HIPEC) is an effective treatment for PM of non-gynecologic origin and, recently, epithelial ovarian cancer. We evaluated the feasibility of CRS/HIPEC in the management of PM from rare ovarian malignancies and report postoperative outcomes on these patients.

**Methods:**

A retrospective review of a single center, prospective database (1994–2021) was performed to identify patients with rare ovarian malignancies treated with CRS/HIPEC. Clavien-Dindo 90-day morbidity/mortality and Kaplan–Meier overall (OS) and progression-free survival (PFS) were analyzed.

**Results:**

Of 44 patients identified, 28 underwent CRS/HIPEC. Six were aborted due to extensive disease. Histologic subtypes included: clear cell (5/28, 17.9 %), endometrioid (5/28, 17.9 %), granulosa cell (3/28, 10.7 %), low-grade serous (6/28, 21.4 %), mesonephric (1/28, 3.6 %), mucinous (6/28, 21.4 %), and small cell (2/28, 7.1 %) carcinomas. Eight (28.6 %) patients had primary and 20 (71.4 %) had recurrent disease. Median peritoneal cancer index (PCI) was 21 (IQR: 6–29). Complete cytoreduction (<2.5 mm residual disease) was achieved in 27/28 (96.4 %). Grade III/IV complications occurred in 9/28 (32.1 %) with one (3.6 %) mortality. After a median follow-up of 65.8 months, 20 patients were alive. Five-year OS and PFS were 68.5 and 52.6 %, respectively.

**Conclusions:**

In patients with PM from rare ovarian malignancies, CRS/HIPEC is feasible and has an acceptable safety profile. Longer follow-up and multicenter trials are needed.

## Introduction

Similar to more common ovarian subtypes, some rare ovarian malignancies (incidence <6/100,000 cases/year) can spread to the peritoneum [1, 2], which leads to treatment challenges, as well as significant morbidity and poor survival. There is no clear data regarding management of peritoneal metastases (PM) in rare ovarian malignancies and, despite representing 50 % of all gynecologic malignancies [3], [4], [5], these patients are under-represented in most cancer trials. The current standard of care for ovarian cancer with PM, including rare subtypes, is cytoreductive surgery (CRS) that aims for removal of all macroscopic disease and systemic chemotherapy. However, the high recurrence rates after CRS and limited effect of systemic chemotherapy in low vascularized peritoneal lesions warrants the use of new strategies that can provide better loco-regional control.

Delivering high concentrations of hyperthermic intraperitoneal chemotherapy (HIPEC) to the peritoneal cavity after a complete CRS successfully controls PM in non-gynecologic malignancies, such as appendix and mesothelioma [6, 7]. Peritoneal spread of ovarian cancer can display a similar clinical presentation to these peritoneal surface malignancies. This is especially true in some rare ovarian tumors (mucinous ovarian cancer), where voluminous, mucinous ascites, and poor chemotherapy response is observed [8], [9], [10]. In line with this, evidence shows that epithelial ovarian cancer can also benefit from CRS/HIPEC by prolonging time to recurrence [11]. Thus, CRS/HIPEC may also be useful in rare ovarian subtypes given the similar presentation.

Limited knowledge about these rare tumors and low number of annual cases makes it difficult to design a prospective study and analyze different treatments. Not surprisingly, randomized control trials are lacking and most treatment decisions are drawn from experience with more common ovarian subtypes or tumors with similar pathobiology [5, 12], [13], [14]. In this sense, any knowledge about their course and response to oncologic treatments in advanced stages serves as useful information. Considering the benefit of CRS/HIPEC in the treatment of PM and its safety profile, we hypothesize that patients with PM from rare ovarian malignancies can safely undergo CRS/HIPEC, which may delay disease progression. We evaluated the feasibility of CRS/HIPEC in the management of PM from rare ovarian malignancies and reported the postoperative outcomes for these patients treated at a high-volume center.

## Subjects and methods

### Patient selection

A retrospective review (1994–2021) of a prospective database of >1,000 patients with peritoneal dissemination from different primaries (appendix, colon, mesothelioma, and ovarian cancer) treated with CRS/HIPEC at a single-, high-volume peritoneal surface malignancy center was performed. Patients with newly diagnosed or recurrent PM (The International Federation of Gynecology and Obstetrics [FIGO] stage III/IV) of primary peritoneal, fallopian tube, or ovarian origin with rare histology who underwent CRS/HIPEC at our institution with ≥3 months follow-up were included. All patients were classified according to the World Health Organization (WHO) and re-staged according to FIGO 2014 criteria [13]. Histopathology reports from previous biopsies or surgeries were reviewed and confirmed by CRS/HIPEC experienced, board certified pathologists. Tumors were defined as rare if recording an incidence of less than six cases per 100,000 annually [3], and included non-high grade serous (HGS) tumors, sex-cord, and germ cell tumors. Carcinosarcomas, complex and rare tumors that involve both cellular components of epithelium and mesenchyme, were excluded from this study and its outcomes were analyzed separately [15, 16]. Patients who had an aborted HIPEC were excluded from the post-HIPEC morbidity/mortality analysis.

### CRS/HIPEC and data collection

CRS was performed as previously described [17] by both a gynecological and surgical oncologist. Selection criteria for surgical candidates was extrapolated from standard criteria of common indications for CRS/HIPEC. In brief, patients with an Eastern Cooperative Oncology Group (ECOG) performance status ≤2, adequate bone marrow, renal, hepatic function, and blood coagulation parameters, a sufficient support system, and in whom achieving a complete cytoreduction was feasible were deemed surgical candidates [17]. Certain imaging findings (CT scan or PET-CT) were taken into account for determining the feasibility of a complete cytoreduction. These included disease extension outside the peritoneal cavity, biliary obstruction, foreshortened mesentery, diffuse carcinomatosis on the small intestinal loops and at the diaphragmatic level, and/or liver parenchymal involvement [17]. Of note, no specific PCI cutoff was used to determine surgical eligibility.

Tumor burden, measured with the peritoneal cancer index (PCI), was assessed at exploration, with PCI≥20 considered high tumor burden [18]. Major resections, defined as surgical procedures that independently require an inpatient admission, were performed as needed to achieve a complete cytoreduction [19]. These included splenectomy, diaphragmatic peritonectomies, diaphragmatic resection, portal dissection, liver resection, gastrectomy, omentectomy, small bowel resection, colectomy, low anterior resection, hysterectomy, salpingo-oophorectomy, cystectomy, vaginectomy, and abdominal or retroperitoneal lymphadenectomy. Completeness of cytoreduction (CC) score was recorded after tumor resection as CC-0 (no residual disease), CC-1 (residual tumor <2.5 mm), CC-2 (residual tumor 2.5–25 mm), and CC-3 (residual tumor >25 mm) [20]. Considering the depth of tissue penetration by cytotoxic drugs, tumor deposits of <2.5 mm (CC-0/1) were considered complete cytoreductions [21]. A complete CRS was aimed for every patient. Following CRS, HIPEC was performed with carboplatin (800 mg/m^2^), cisplatin (50 mg/m^2^) + doxorubicin (15 mg/m^2^), cisplatin + paclitaxel (70 mg/m^2^), melphalan (50 mg/m^2^), or mitomycin-C (40 mg) for 90 min at a temperature of 41–43 °C. Chemotherapeutic agents were selected by consensus between gynecological, surgical, and medical oncologists, based on histology and previous chemotherapy response. In the case of an incomplete CRS, HIPEC perfusion was performed as a palliative procedure. All anastomoses were performed after perfusion. CRS/HIPEC was performed using both the open technique (coliseum), which was the first method used by our group, and the closed technique. Postoperative complications, including readmissions, were graded according to the Clavien–Dindo classification for 90 days after surgery [22]. Grade III/IV complications were considered major.

The Institutional Review Board approved this study. Consent was obtained from all patients as part of an ongoing observational cohort study. Patients were aware of the experimental nature of the treatment and counseled on the availability of other therapies.

### Postoperative care and follow-up

Patients were transferred to the intensive care unit (ICU) during the first 24 h after surgery and then to the inpatient floor when clinically stable. Length of ICU and hospital stay (LOS) was recorded for each patient. Postoperative follow-up occurred at two and four weeks post-discharge, every three months for two years, and then every six months thereafter. Follow-up included physical exam, tumor markers, and CT/PET-CT. Tumor recurrence was diagnosed based on physical exam, rising tumor markers, imaging studies, biopsy results, and/or clinical presentation.

### Statistical analysis

Statistical analysis was performed using SPSS V.23.0. Medians and interquartile ranges (IQR) were used to describe continuous variables not normally distributed. Categorical variables are presented as counts and proportions. Progression-free survival (PFS) was defined as the time in months from CRS/HIPEC to the date of tumor recurrence or death, whichever occurred first. Overall survival (OS) was calculated from the date of CRS/HIPEC to date of death or last follow up. PFS and OS were calculated using the Kaplan-Meier method and compared using the Log-rank test.

## Results

### Patients and tumor types

**Figure 1:**
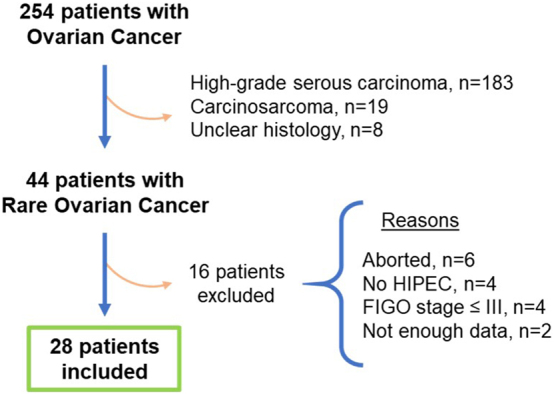
Patient selection flow-chart (1994–2021)

In total, 44 patients with rare ovarian tumors were identified. Of these, 28 were included for analysis. We excluded six patients in which CRS/HIPEC was aborted due to extensive disease (n=1 granulosa cell, n=3 low-grade serous, n=2 mucinous), four patients who only underwent CRS, and six for other reasons (n=4 stage, n=2 amount of data available) (Figure 1). Seven histologic subtypes were identified, including clear cell (5/28, 17.9 %), endometrioid (5/28, 17.9 %), granulosa cell (3/28, 10.7 %), low-grade serous (6/28, 21.4 %), mesonephric (1/28, 3.6 %), mucinous (6/28, 21.4 %), and small cell (2/28, 7.1 %) carcinomas. The location of tumor origin was tubo-ovarian in 25 (89.3 %) patients and primary peritoneal in three (10.7 %) patients. Twenty-three (82.1 %) patients were classified as FIGO stage III and five (17.9 %) as stage IV. Median age at diagnosis was 54 (IQR: 47–57) years. Eight (28.6 %) patients had primary and 20 (71.4 %) had recurrent disease. Median time from diagnosis to CRS/HIPEC was 23.2 (IQR: 1.7–48.0) months. All patients in the primary group were treated upfront, with no interval cases. In the recurrent group, all (20/20, 100 %) patients had prior surgery and 14/20 (70.0 %) had prior chemotherapy (Table 1).

**Table 1:** CRS/HIPEC patient characteristics and postoperative outcomes.

Pt ID	Age at CRS/HIPEC, years	Primary tumor/location	FIGO stage	PCI	CC score	HIPEC agent	Adjuvant chemotherapy (number of cycles)	Site of first recurrence	Status	PFS, months	OS, months
1	44	CCC/PP	IIIC	29	CC-0	Carboplatin	Carboplatin+Paclitaxel (6)	–	NED	102.1	102.1
2	56	CCC/TO	IIIA1 (ii)	6	CC-0	Cisplatin+Doxorubicin	Carboplatin+Paclitaxel (6)	–	NED	7.4	7.4
3	52	CCC/TO	IVB	3	CC-0	Melphalan	–	–	NED	50.0	50.0
4	51	CCC/TO	IIIA1(i)	4	CC-0	Carboplatin	–	–	NED	2.1	2.1
5	58	CCC/TO	IIIC	32	CC-0	Melphalan	–	Liver capsule and parenchyma	AWD	6.0	7.8
6	54	EC/TO	IVB	27	CC-1	Melphalan	Carboplatin+Paclitaxel (6)	–	NED	81.5	81.5
7	55	EC/TO	IIIC	24	CC-3	Carboplatin	Carboplatin+Paclitaxel (15)	Not applicable	DOD	0.0^a^	16.4
8	49	EC/TO	IIIB	3	CC-0	Cisplatin+Doxorubicin	Carboplatin+ cyclophosphamide (4)	–	NED	227.7	227.7
9	63	EC/TO	IIIC	10	CC-0	Melphalan	–	Abdominal wall	AWD	74.6	103.0
10	56	EC/TO	IVB	3	CC-0	Melphalan	Niraparib	Bladder lumen	AWD	39.3	62.4
11	51	GCT/TO	IIIA2	10	CC-0	Carboplatin	–	–	NED	31.7	31.7
12	73	GCT/TO	IIIC	5	CC-0	Cisplatin+Doxorubicin	–	–	NED	9.7	9.7
13	76	GCT/TO	IIIC	21	CC-0	Melphalan	–	–	DOC^b^	0.5	0.5
14	58	LGS/PP	IIIC	15	CC-0	Carboplatin	–	Abdominal muscle	DOC	40.8	68.3
15	62	LGS/TO	IIIC	24	CC-0	Carboplatin	Carboplatin+Docetaxel (3)	–	NED	64.9	64.9
16	58	LGS/TO	IIIC	30	CC-0	Carboplatin	Carboplatin+Paclitaxel (6); letrozole	Iliac LN	AWD	23.7	26.2
17	36	LGS/TO	IIIC	34	CC-0	Melphalan	Letrozole/Anastrazole	Abdominal wall	DOD	17.99	72.8
18	56	LGS/PP	IIIC	24	CC-0	Melphalan	Gemcitabine+Carboplatin (2); Gemcitabine+Carboplatin+ bevacizumab (4)	Bladder wall, gastro-hepatic ligament and pelvis	AWD	10.1	73.4
19	59	LGS/TO	IIIC	29	CC-0	Cisplatin+Paclitaxel	IP carboplatin (6)	Liver capsule	DOD	18.4	30.4
20	54	MA/TO	IVB	29	CC-0	Carboplatin	Carboplatin+Docetaxel (6)	Pleura and pericardial LN	AWD	9.3	30.9
21	50	MCa/TO	IIIA2	5	CC-0	Mitomycin-C	–	–	NED	142.5	142.5
22	61	MCa/TO	IIIA2	9	CC-0	Mitomycin-C	–	–	NED	135.9	135.9
23	49	MCa/TO	IIIA2	8	CC-0	Mitomycin-C	–	–	NED	103.7	103.7
24	71	MCa/TO	IIIA2	6	CC-0	Mitomycin-C	–	–	DOC	26.02	37.2
25	63	MCa/TO	IIIB	27	CC-0	Mitomycin-C	–	–	NED	36.8	36.8
26	42	MC/TO	IIIC	22	CC-0	Mitomycin-C	–	Liver parenchyma	DOD	4.3	7.3
27	74	SCC/TO	IIIC	31	CC-0	Carboplatin	Carboplatin+Gemcitabine (6)	Manubrium and liver capsule	DOD	14.3	50.1
28	37	SCC/TO	IVB	35	CC-0	Melphalan	–	Ascites	DOD	0.3	1.4

^a^Patient with residual disease (>2.5 mm) after surgery. ^b^Postoperative death after CRS/HIPEC due to septic shock secondary to ischemic bowel disease. Patients with primary disease treated with primary CRS/HIPEC are highlighted in gray. AWD, alive with disease; CCC, clear cell carcinoma; CC-score, completeness of cytoreduction score; CRS/HIPEC, cytoreduction with hyperthermic intraperitoneal chemotherapy; DOD, dead of disease; EC, endometrioid carcinoma; GCT, granulosa cell tumor; LGS, low-grade serous; LN, lymph node; MA, mesonephric adenocarcinoma; MC, mucinous carcinoma; MCa, mucinous cystadenoma; NA, not available; NED, no evidence of disease; PCI, peritoneal carcinomatosis index; PP, primary peritoneal; Pt, patient; SCC, small cell carcinoma; TO, tubo-ovarian.

### Operative outcomes

Median age at CRS/HIPEC was 56 (IQR: 50–62) years. Median PCI score was 21 (IQR: 6–29). Median number of major resections was five (IQR: 2–7) and median estimated blood loss was 500.0 (IQR: 325.0–1,387.5) mL. Lymph nodes were submitted for pathological analysis in 27/28 (96.4 %) patients and 11 (40.7 %) of these patients had lymph node involvement. Median length of surgery was 532 (IQR: 411–596) minutes. Complete cytoreduction (CC-0/1) was achieved in 27/28 (96.4 %) patients who underwent CRS/HIPEC. One (3.5 %) patient had residual disease (CC-3) and received palliative HIPEC. HIPEC agents were carboplatin (n=9, 32.1 %), cisplatin + doxorubicin (n=3, 10.7 %), cisplatin + paclitaxel (n=1, 3.6 %), melphalan (n=9, 32.1 %), and mitomycin-C (n=6, 21.5 %) (Table 1). HIPEC was performed using the open (coliseum) method in two (7.1 %) patients and the closed technique in 26 (92.9 %) patients. In one patient, HIPEC was shortened to 45 min due to persistent hypotension.

### Morbidity and mortality after CRS/HIPEC

Median length of hospital stay was nine (IQR: 8–11) days. The readmission rate was 7/28 (25.0 %). At least one complication occurred in 24 (85.7 %) patients. Grade I complications occurred in 12/28 (50.0 %) patients (anemia, thrombocytopenia, diarrhea, and leukopenia). Grade II complications, particularly anemia requiring blood transfusion (13/19, 68.4 %), were the most common type (19/28, 67.9 %). Major complications occurred in 9/28 (32.1 %) patients, with 4/9 (44.4 %) patients presenting with more than one major complication. Major complications included intraoperative ureteral injury (n=1), pyelonephritis (n=1), recurrent ascites (n=2), left upper quadrant sterile collections (n=3), pelvic abscess (n=1), wound dehiscence (n=2), pancreatic leak (n=1), enterocutaneous fistula (n=1), vaginal fistula (n=1), gastrointestinal obstruction (n=1), and septic shock (n=2). One (3.6 %) 90-day mortality occurred on postoperative day 14 due to septic shock secondary to ischemic bowel (Table 2).

**Table 2:** Clavien–Dindo 90-day complications after CRS/HIPEC

System	Complication	Clavien–Dindo classification			
		I	II	III	IV
Cardiovascular	Arrhythmia	0	2	0	0
	Hypotension	0	3	0	0
Genitourinary	Pyelonephritis	0	0	1	0
	Ureteral injury	0	0	1	0
	Vaginal fistula	0	0	1	0
Gastrointestinal	Ascites	0	0	2	0
	Diarrhea	2	0	0	0
	Enterocutaneous fistula	0	0	1	0
	Gastrointestinal obstruction	0	0	0	1
	Left upper quadrant collection	0	0	3	0
	Nausea/vomiting	0	4	0	0
	Pancreatic leak	0	0	1	0
	Wound dehiscence	0	0	1	0
Hematological	Anemia	9	13	0	0
	Coagulopathy	0	1	0	0
	Leukopenia	2	4	0	0
	Thrombocytopenia	4	3	0	0
Infectious	Pelvic abscess	0	1	0	0
	Septic shock	0	0	0	2
	Urinary tract infection	0	3	0	0
	Vaginal yeast infection	0	1	0	0
Neurological	Confusion	0	2	0	0
Pulmonary	Pneumonia	0	1	0	0
	Pleural effusion	0	1	0	0
	Respiratory distress	0	1	0	0
Renal	Renal failure	0	1	0	0
Skin	Wound dehiscence	0	0	1	0
	Wound infection	0	2	0	0

Complications of any grade occurred in 24/28 patients. Of these, 19/24 patients presented with more than one complication of the same or different grade. Major complications (grade III/IV) occurred in 9/28 patients.

### Survival and recurrence

**Figure 2:**
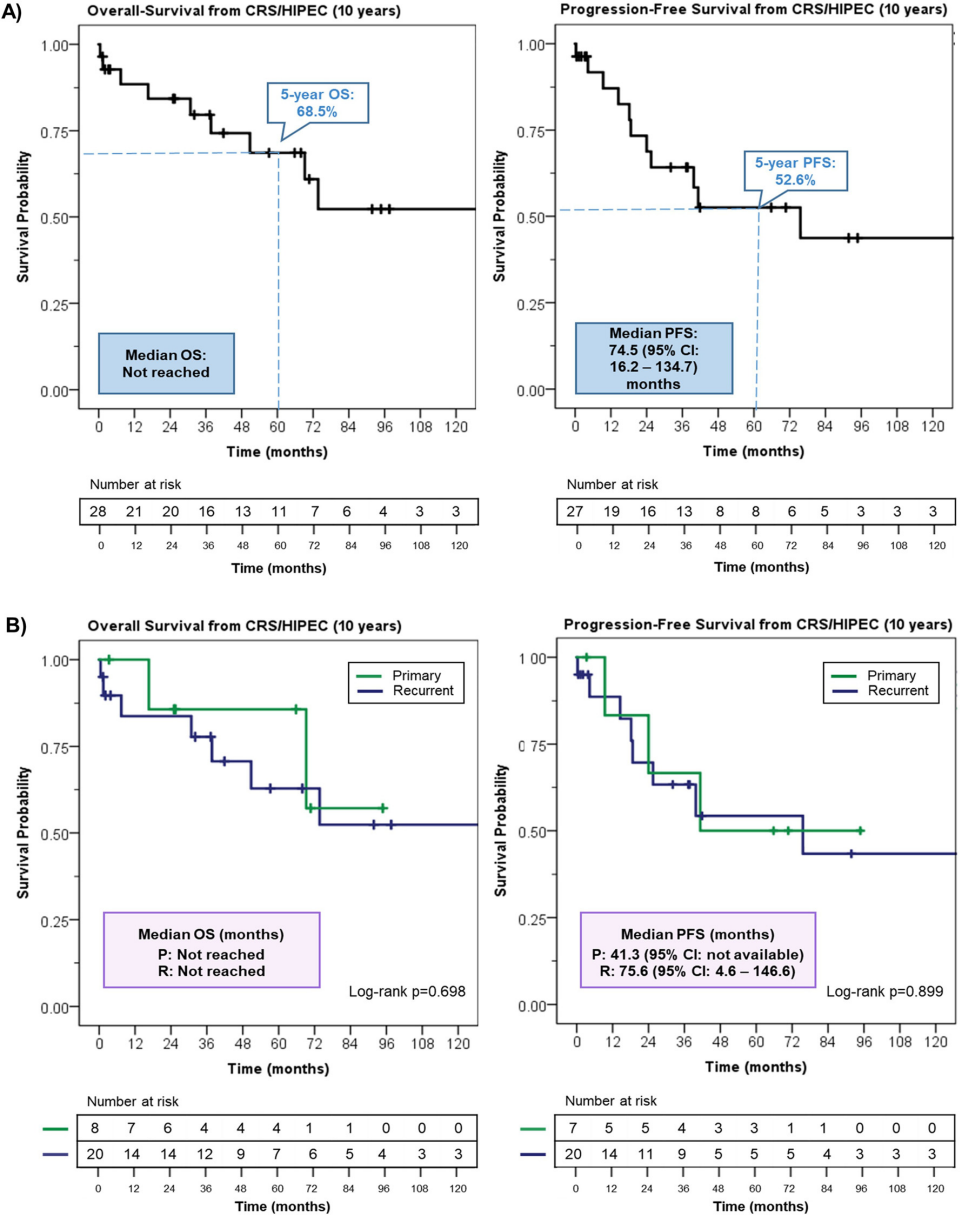
Survival outcomes after CRS/HIPEC.

After CRS/HIPEC, 15/28 (53.6 %) patients received adjuvant systemic chemotherapy (Table 1). Carboplatin + paclitaxel was the most common regimen with a median of six cycles (range: 6–15). Median follow-up after CRS/HIPEC was 65.8 (95 % confidence interval (CI): 43.6–88.0) months, with a two-, five-, and ten-year follow-up of 85.4 , 54.1, and 19.4 %, respectively. At last follow-up, 12/28 (42.9 %) patients had disease recurrence. Sites of recurrence included intraperitoneal only in 3/12 (25.0 %) patients, extraperitoneal only in 6/12 (50.0 %) patients, and both in 3/12 (25.0 %) patients (Table 1). Median PFS was 75.6 months (95% CI: 16.2–134.7) with a two-, five-, and ten-year PFS of 68.8 , 52.6, and 43.8 %, respectively (Figure 2A). Median OS was not reached, with a two-, five-, and ten-year OS of 88.4 , 68.5, and 52.2 %, respectively (Figure 2A). No differences in OS (p=0.698) or PFS (p=0.899) were observed between primary and recurrent patients (Figure 2B).

## Discussion

Rare ovarian cancers include a variety of tumors with diverse biology. Given the high recurrence rates and low chemotherapy response for some subtypes (i.e. clear cell and small cell carcinomas), there is an unmet need for additional treatment options [23]. Since rare ovarian malignancies can present with extensive, often debilitating loco-regional PM, the addition of HIPEC to CRS poses as a suitable option for peritoneal disease control and treatment in these select patients. We present perioperative and long-term outcomes of a cohort of patients with PM from rare ovarian tumors treated with CRS/HIPEC, including seven histologic subtypes (clear cell, endometrioid, low-grade serous, mesonephric, mucinous, granulosa cell, and small cell carcinomas).

For all subtypes, achieving a complete cytoreduction remains a major prognostic factor for ovarian cancer. There is substantial evidence that optimal survival outcomes after CRS are achieved in ovarian cancer patients with little or no residual disease at the completion of surgery [24, 25]. Maximal efforts should be made to remove all visible disease. Therefore, the ability to achieve a complete cytoreduction is paramount in patient selection for CRS/HIPEC. In our cohort, 27/28 (96.4 %) patients who underwent CRS/HIPEC had minimal residual disease (tumors <2.5 mm) after CRS. These results are similar to those reported in ovarian cancer trials evaluating CRS/HIPEC [11, 26], where minimal residual disease was left in 87 % of patients, demonstrating that a CC-0/1 can also be accomplished for advanced rare ovarian tumors.

Even after a complete CRS, most patients with ovarian cancer (up to 60 %) recur in the peritoneal cavity [27, 28]. HIPEC can target residual microscopic disease left behind after CRS, improving the quality of CRS and assisting in the delivery of high-dose chemotherapy directly to the peritoneum. These benefits are evidenced by a significantly lower incidence of peritoneal recurrences in patients with ovarian cancer treated with CRS/HIPEC compared to those treated with CRS alone [29], [30], [31], suggesting that adding HIPEC to interval CRS improves intraperitoneal disease control in HGS ovarian cancer [32]. In our study, less than 50 % of patients had disease recurrence after CRS/HIPEC, with higher rates of extra-vs. intra-peritoneal recurrences (50.0 vs. 25.0 %). This suggests that HIPEC can also provide loco-regional disease control in rare ovarian tumors.

Understandably, CRS/HIPEC implementation has been limited by concerns around its safety profile. As an extensive procedure, CRS/HIPEC can pose significant risks for postoperative morbidity and mortality. However, when performed at experienced centers and following defined guidelines, significant surgical trauma can be minimized [33, 34]. Our cohort had an acceptable major complication (32.1 %) and mortality rate (3.6 %), similar to that reported for CRS alone and in previous HIPEC trials [11, 26, 33, 35, 36]. A single intra-procedural complication of hypotension occurred, which resulted in a shortened HIPEC perfusion. The most common complication was anemia, which responded to blood transfusion. Accordingly, CRS/HIPEC has an acceptable safety profile in rare ovarian tumors, with results similar to that reported for HGS ovarian cancer.

No prospective trials have evaluated the potential benefit of adding HIPEC to the treatment of rare ovarian tumors. Both of the phase-III clinical trials that demonstrated a survival benefit of adding HIPEC for advanced ovarian cancer treatment only included a few patients with rare ovarian malignancies, but no histology specific outcomes were reported due to limited sample size [11, 26]. Use of HIPEC in rare ovarian tumors has been mostly documented in retrospective studies. These studies mainly consisted of heterogeneous cohorts including non-gynecologic tumors, analyzed all together and with limited information by tumor type [10, 37]. The PSOGI Working Group (2018) and INDEPSO (2022) have reported two cohorts of patients with rare ovarian cancer treated with CRS/HIPEC [2, 9]. Both demonstrated an acceptable morbidity (major complication rate: 42.2 and 15.8 %, respectively) and feasibility for achieving complete CRS (CC-0/1: 92.5 and 81.2 %, respectively). No definite conclusions for OS and PFS were possible given the limited data by tumor subtype and diverse treatment groups, including multiple perfusion agents. However, the PSOGI Working Group observed significant differences in PFS within rare cancer subtypes, showing best results for mucinous tumors. Although results from these studies and ours are limited, the addition of HIPEC to treat advanced patients with rare ovarian cancer warrants further consideration and encourages the conduction of more systematic and multi-center investigations.

The main strengths of our study are the rigor of our data collection, a low lost to follow-up rate (0 %) in under-reported histologic subtypes, experience as a high-volume peritoneal surface malignancy center, and strict long-term follow-up. We acknowledge that our study has some limitations related to the retrospective nature, inclusion of patients treated over >20 year span (representing variations in patient selection, management and surveillance), small sample size without a comparison group, and the high number of rare ovarian histologies resulting in the use of multiple HIPEC agents and adjuvant chemotherapy recommendations. Nonetheless, this study provides evidence of an available treatment option that is safe and can provide long-term outcomes in select patients with PM from rare ovarian malignancies.

Our study confirms that HIPEC can be safely used to treat PM of rare ovarian tumors after achieving a complete CRS (CC-0/1) for both primary and recurrent patients. The long PFS (median: 75.6 months) and low percentage of peritoneal recurrences (25.0 %) support the hypothesis that CRS/HIPEC may provide loco-regional disease control; however, questions remain around which HIPEC agent should be used and the true survival benefit of CRS/HIPEC for each histology.

## Conclusions

We demonstrated that CRS/HIPEC is feasible and has an acceptable safety profile for PM originating from rare ovarian tumors, with the possibility of providing local tumor control. Longer follow-up and multicenter collaborative trials are needed to address this hypothesis and define survival outcomes for each histology.
